# Young-IFSO Endoscopy Training and Education Survey

**DOI:** 10.1007/s11695-025-07915-4

**Published:** 2025-06-03

**Authors:** Daniel Moritz Felsenreich, Anna Carolina Batista Dantas, Shahab Shahabi, Tamer N. Abdelbaki, Roxanna Zakeri, Mostafa E. Nagy, Daniel Gero, Elena Ruiz-Úcar, Zhi-Yong Dong, Wah Yang, Megan Jenkins, Sonja Chiappetta, on behalf of Young-IFSO Collaborative Group

**Affiliations:** 1 Division of Visceral Surgery, Department of General Surgery, Medical University of Vienna, Vienna, Austria; 2 Departamento de Cirurgia, Universidade Federal do Rio Grande do Norte (UFRN), Natal, Brazil; 3 Department of Surgery, Minimally Invasive Surgery Research Center, Division of Minimally Invasive and Bariatric Surgery, Iran University of Medical Sciences, Tehran, Islamic Republic of Iran; 4 Alexandria University Faculty of Medicine, Alexandria, Egypt; 5 Department of Upper GI Surgery, University College London Hospital NHS Foundation Trust, London, United Kingdom; 6 General Surgery Department, Ain Shams University, Cairo, Egypt; 7 Department of Surgery and Transplantation, University Hospital Zurich, University of Zurich, Zurich, Switzerland; 8 Metabolic and Endocrine Unit, General and Digestive Surgery Department, Fuenlabrada University Hospital, Rey Juan Carlos University, Madrid, Spain; 9 Department of Metabolic and Bariatric Surgery, The First Affiliated Hospital of Jinan University, Guangzhou, China; 10 Department of Surgery, Assistant Professor, NYU Langone Health, NY, New York, United States; 11 Department of General Surgery, Center of Excellence Bariatric and Metabolic Surgery, Ospedale Evangelico Betania, Naples, Italy

**Keywords:** Endoscopy, Endoscopic sleeve gastroplasty, Transoral outlet reduction, Endoscopic diagnostic, Endoscopic treatment, Intragastric balloon

## Abstract

**Background:**

With the increasing complexity of metabolic and bariatric surgery (MBS) techniques, the integration of endoscopic modalities has become indispensable in both pre-, intra-, and postoperative management. This survey aimed to evaluate the current landscape of endoscopic training, practice patterns, and proficiency among early-career surgeons.

**Methods:**

Cross-sectional study based on online survey voluntary and open to all Young-IFSO members (surgeons aged ≤ 45 years) between September and December 2024. The questionnaire addressed participants’ socio-demographic characteristics, professional background, and details regarding their endoscopic training and practice. Particular emphasis was placed on current engagement in endoscopic management of MBS-related complications and endoscopic bariatric therapies (EBTs).

**Results:**

A total of 273 respondents from 49 countries completed the survey. Endoscopic training was included in the general surgery curriculum for half of the participants, yet 33.8% do not perform endoscopy in current practice. Nearly half (46.9%) reported lacking confidence in managing MBS complications endoscopically. Although EBTs are available in half of the respondents’ institutions, only 23.9% perform these procedures themselves.

**Conclusion:**

This Young-IFSO survey highlights the limited endoscopic involvement of early-career metabolic and bariatric surgeons and the need for structured training to meet the demands of modern MBS.

**Supplementary Information:**

The online version contains supplementary material available at 10.1007/s11695-025-07915-4.

## Introduction

Metabolic and bariatric surgery (MBS) has evolved into a cornerstone of obesity treatment [1]. With the advancement of surgical techniques, integration of endoscopic modalities has become indispensable, as endoscopy plays a pivotal role in preoperative anatomical assessment, intraoperative quality control, and management of postoperative complications [2, 3]. Furthermore, endoscopic bariatric therapies (EBTs), including intragastric balloon (IGB) placement, endoscopic sleeve gastroplasty (ESG), and transoral outlet reduction (TORe), have emerged as minimally invasive alternatives expanding treatment options for patients across the obesity disease spectrum [3].

Despite its critical role, endoscopic proficiency among early-career bariatric surgeons remains heterogeneous globally. A previous Young-IFSO (International Federation for the Surgery of Obesity and Metabolic Disorders) MBS Training and Education Survey highlighted significant gaps in standardized endoscopic training during surgical residency, reporting that less than half of respondents performed diagnostic upper endoscopy independently, while only 30% received training in endoscopic MBS-related complication management [4]. These disparities are further compounded by regional variations in regulatory frameworks, resource availability, institutional support, and access to advanced endoscopic technologies.

Young-IFSO, a global consortium dedicated to advancing education and research in MBS, has prioritized addressing these gaps through international collaboration and advocacy [5]. However, comprehensive data on the current state of endoscopic training and practice among MBS professionals remains scarce. This survey aimed to evaluate the current landscape of endoscopic training, practice patterns, and proficiency among early-career metabolic and bariatric surgeons.

## Methods

This cross-sectional study was based on an online survey to evaluate endoscopic training, practice patterns, and self-reported proficiency among early-career metabolic and bariatric surgeons worldwide. All members of Young-IFSO, defined as surgeons aged ≤ 45 years, were invited to participate voluntarily via email and social media platforms managed by IFSO, between September and December 2024.

The survey comprised 40 questions in English, covering participants’ sociodemographic characteristics, professional background, and specific aspects of their endoscopic training and clinical practice (Supplementary Material). The questionnaire was administered using SurveyMonkey™ (San Mateo, CA, USA), and submission was only possible once all questions were completed.

Collaborative authorship was offered to respondents who consented to it; however, all data were anonymized prior to analysis. Descriptive statistics and data visualization were performed in Microsoft Excel© Professional Plus 2021. The manuscript was prepared in accordance with the Checklist for Reporting Results of Internet E-Surveys guidelines [6].

## Results

A total of 273 respondents from 49 countries completed the survey. As shown in Table 1, most of our respondents are male (76.9%), metabolic and bariatric surgeons (82.1%), and currently working as consultant surgeon (69.2%).

**Table 1:** Demographic characteristics

	*N* = 273
Sex, *n* (%)	
Male	210 (76.9)
Female	63 (23.1)
Median age, years (IQR)	36 (IQR 32.5–40)
Current position, *n* (%)	
Student	3 (1.1)
Trainee/resident	41 (15.0)
Consultant surgeon	189 (69.2)
Consultant endoscopist	13 (4.8)
Other	27 (9.9)
Specialty, *n* (%)	
Metabolic and bariatric surgeon	224 (82.1)
Endoscopist	13 (4.8)
Other	36 (13.2)
Type of hospital, *n* (%)	
Private	65 (23.8)
Public	62 (22.7)
University	91 (33.3)
Combined private and public	47 (17.2)
Military	1 (0.4)
Other	7 (2.6)

*IQR*, interquartile range

### Endoscopic Training and Experience

**Fig. 1:**
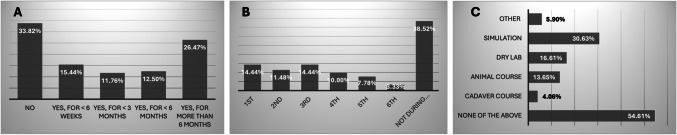
Endoscopic training curriculum during surgical residency (**1 A**), year of education for the first procedure (**1B**), and structure training available at their institution (**1 C**)

Half of the respondents reported having endoscopic education integrated into their general surgery residency curriculum; however, only 26.5% had access to training for more than six months (Fig. 1A). Notably, 30.8% did not perform any endoscopic procedures during residency. Among those who did, the timing of their first endoscopy varied considerably across training stages (Fig. 1B), with a mean age of 30.2 ± 4.2 years. Although 83.4% expressed interest in attending formal upper endoscopy training courses, 56.3% had never participated in one. Some respondents indicated that their current institutions offer structured endoscopic training—primarily through simulation (30.6%), but also via dry labs (16.6%) and animal courses (13.6%) (Fig. 1C).

While 65.0% currently perform endoscopies in clinical practice, the reported annual procedural volume remains low. As detailed in Table 2, only 34.7% and 3.4% perform more than 100 routine and emergency endoscopies per year, respectively. Most notably, despite most of them (71.3%) having an endoscopy tower available in the operating room (OR), only 23.1% routinely perform intraoperative endoscopy (IOE) (e.g., leak test, evaluation of staple line bleeding) at the end of MBS.

**Table 2:** Current endoscopic practice

	*N* (%)
Upper endoscopies (excluding ERCP) performed per year (*N* = 266)	
0	93 (35.0)
1–50	59 (22.2)
51–100	33 (12.4)
101–500	60 (22.6)
501–1000	16 (6.0)
> 1000	5 (1.9)
Lower endoscopies performed per year (*N* = 267)	
0	132 (49.4)
1–50	58 (21.7)
51–100	29 (10.9)
101–500	35 (13.1)
501–1000	10 (3.7)
> 1000	3 (1.1)
Routine endoscopies performed per year (*N* = 265)	
0	93 (35.1)
1–50	50 (18.9)
51–100	30 (11.3)
101–500	71 (26.8)
501–1000	13 (4.9)
> 1000	8 (3.0)
Emergency endoscopies performed per year (*N* = 266)	
0	125 (47.0)
1–50	114 (42.9)
51–100	18 (6.8)
101–500	7 (2.6)
501–1000	1 (0.4)
> 1000	1 (0.4)
ERCP performed per year (*N* = 266)	
0	219 (82.3)
1–50	29 (10.9)
51–100	4 (1.5)
101–500	13 (4.9)
501–1000	0 (0.0)
> 1000	1 (0.4)
Total endoscopies performed during residency (*N* = 266)	
0	82 (30.8)
1–50	77 (28.9)
51–100	15 (5.6)
101–500	60 (22.6)
501–1000	18 (6.8)
> 1000	14 (5.3)

### Endoscopic Management of Postoperative Complications

**Fig. 2:**
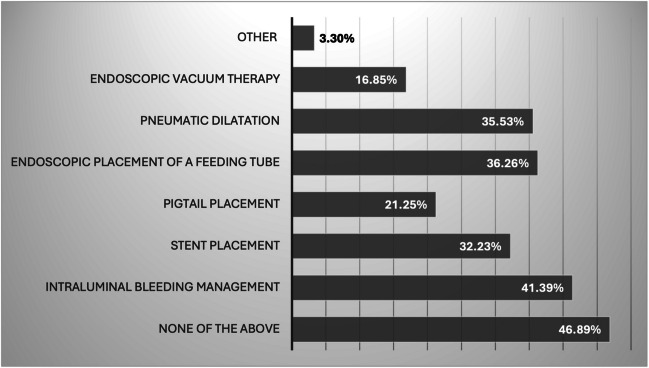
Confidence rate for performing different endoscopic techniques for postoperative complication management

While the majority (89.7%) reported that endoscopic management of MBS-related complications is performed at their hospital, only 34.19% were personally involved in such procedures. As shown in Fig. 2, almost half of our respondents (46.9%) are not confident in performing the most commonly available endoscopic techniques for complication management.

### Endoscopic Bariatric Therapies

Half of our respondents (49.8%) reported that EBTs are routinely performed at their institutions. While 52.2% recommend EBTs as treatment option, only 23.9% perform it by themselves. Intragastric balloon (IGB) placement was the most commonly available procedure (60.7%) although only 34.6% of respondents perform it. Endoscopic sleeve gastroplasty (ESG) was available in 26.4% of institutions and performed independently by 12.5% of our surgeons. Transoral outlet reduction (TORe) following Roux-en-Y gastric bypass (RYGB) was the least available EBTs (19.2%) and performed by just 8.1% of respondents.

## Discussion

This survey aimed to evaluate the current landscape of endoscopic training, practice patterns, and proficiency among early-career metabolic and bariatric surgeons worldwide. Based on responses from 273 Young-IFSO members from 49 countries, we have shown the underrepresentation of structured endoscopic curriculum in surgical training and the low practice volume of endoscopic procedures for obesity treatment and MBS-related complication management.

### Endoscopic Training

Approximately one-third of our respondents reported not performing any endoscopic procedures during their general surgery residency. Among those who did, the procedural volume was generally low, with fewer than 50 cases. This contrasts with data from recent literature, which reports that general surgery residents in the USA perform an average of 130 endoscopic procedures (range: 129–132), a number that has remained stable across most programs since 2010 [7]. However, as these figures reflect only US-based residency programs, our findings underscore the global variability in endoscopic training among surgical trainees and highlight the need for standardized competency benchmarks across different training systems.

There has been increasing interest in bariatric endoscopy training among gastroenterologists, as reflected in the American Gastroenterological Association’s (AGA) White Paper on endoscopic obesity management and the incorporation of EBTs into several gastroenterology fellowship programs [8, 9]. In contrast, structured endoscopic training for general surgeons remains limited. The Society of American Gastrointestinal and Endoscopic Surgeons (SAGES) developed the Fundamentals of Endoscopic Surgery (FES) certification, which has been a requirement of the American Board of Surgery (ABS) since 2018; however, its core curriculum does not include didactic content related to EBTs or MBS [10, 11]. Moreover, no standardized endoscopic training curriculum has yet been formally recommended by professional societies dedicated to metabolic and bariatric surgery.

Finally, although only half of the respondents had attended at least one upper endoscopy course, the vast majority (80%) expressed a desire to do so. Similar findings were reported in a survey conducted by the United Kingdom Roux Group, in which 81.7% of respondents agreed that proficiency in diagnostic endoscopy should be a minimum requirement for obtaining the Certificate of Completion of Training (CCT) in the UK [12]. While access to endoscopic training varies significantly across different regions, our findings reflect a clear expectation and a strong interest in acquiring diagnostic and therapeutic endoscopy skills among young metabolic and bariatric surgeons.

### Endoscopic Practice and Experience

Our survey results revealed significant variability in the frequency of upper and lower endoscopies, reflecting diverse practices in endoscopic procedures among metabolic and bariatric surgeons. Meanwhile, data from the European Association of Endoscopic Surgeons (EAES) indicate that only 10.5% (108 out of 1486) of general surgeons perform more than 100 endoscopies annually [13]. In contrast, 30.5% of our respondents reported performing over 100 endoscopic procedures per year, suggesting that MBS subspeciality may place greater emphasis on endoscopic practice. This trend may reflect the fact that surgeons tend to prioritize operative procedures in their clinical practice, while advanced endoscopic interventions are more commonly performed by specialized gastroenterologists. This division of roles can be influenced by variations in training pathways, healthcare system structures, reimbursement models, and regulatory frameworks across different countries [14, 15].

Intraoperative endoscopy (IOE) is routinely performed by less than 25% of our respondents. While this aligns with current literature based on the American College of Surgeons National Surgical Quality Improvement Program (ACS-NSQIP) showing 17.9% and 19.7% rates of IOE following sleeve gastrectomy and RYGB, respectively [16]; this low adoption rate cannot be attributed to a lack of resources, as 71% of our respondents reported the availability of an endoscopy tower in the OR, nor to insufficient evidence, as IOE has been shown to significantly reduce early postoperative complications after SG [16].

Despite all of the efforts to increase endoscopic practice by surgeons that have been generated by IFSO, SAGES, ASMBS (American Society for Metabolic and Bariatric Surgery), EAES, ACS, ABS, and various other professional and commercial interests, it seems that even though many surgeons were trained in endoscopy, they still are not participating in using these techniques to benefit their patients.

### Endoscopic Management of Postoperative Complications

Bariatric endoscopy has evolved since the 1980 s as a minimally invasive option for managing postoperative complications following MBS, fostering ongoing multidisciplinary collaboration between surgeons and endoscopists [17]. It is associated with low morbidity and can serve both as a definitive treatment and as a bridge to surgery [17, 18]. Despite its benefits, only one-third of our respondents currently perform these procedures, in line with a previous IFSO survey on MBS training and education [4]. Additionally, nearly half (46.9%) reported lacking confidence in performing the most commonly available endoscopic techniques for complication management. This gap may reflect the increasing specialization of bariatric endoscopy among dedicated endoscopists, along with the rapid evolution of techniques and devices in the field. These advances demand ongoing education and access to appropriate equipment, which can be particularly challenging in a globally heterogeneous context, especially in limited-resource settings [9, 17].

### Endoscopic Bariatric Therapies

Endoscopic bariatric therapies (EBTs) comprise a range of flexible endoscopic techniques used in both primary obesity treatment and revisional procedures following metabolic and bariatric surgery [19]. While approximately half of our respondents reported having EBTs available at their institutions and actively recommending its use, fewer than 25% performed these procedures themselves. This limited adoption likely reflects the global absence of standardized training programs. In addition to the cognitive and technical demands of EBTs, barriers such as inadequate infrastructure, limited personnel, restricted access to technology, and a shortage of qualified educators further hinder the establishment and dissemination of effective training pathways [9, 20].

Additionally, the lack of long-term evidence, high costs, and regulatory aspects may limit the availability and adoption of newer EBTs devices [20, 21]. Our findings reveal a decline in performance rates for more recent EBTs procedures, both at respondents’ hospitals and by themselves. Specifically, respondents declared performing IGB, ESG, and TORe at rates of 34.6%, 12.5%, and 8.1%, respectively. Among these, IGB remains the most widely used procedure in clinical practice, supported by three FDA-approved devices and over ten prospective trials with 12-month follow-up data [21].

In 2024, The IFSO Bariatric Endoscopy Committee endorsed ESG as an effective and valuable treatment for obesity, following a comprehensive systematic review and meta-analysis. The committee concluded that ESG is particularly beneficial for patients with class I and II obesity, as well as for select patients with class III obesity who are not suitable candidates for MBS [22]. Additionally, a 2020 systematic review and meta-analysis highlighted TORe as a safe and technically feasible endoscopic option for managing weight recurrence following RYGB [23].

This study is limited by its design as an international online survey, which may introduce heterogeneity due to global disparities in endoscopic training, practice patterns, and proficiency among early-career surgeons. Additionally, selection bias is possible given the specific Young-IFSO population and the voluntary nature of participation. The integration of endoscopy into MBS practice requires a complex skill set that includes diagnostic and therapeutic capabilities, as well as interdisciplinary collaboration. Our findings nevertheless reveal significant gaps in structured endoscopic training and underscore the need for coordinated efforts by educators, institutions, and professional societies to advance and standardize educational pathways in this evolving field.

## Conclusion

This Young-IFSO endoscopy survey highlights the limited integration of structured endoscopic curricula in surgical training and the low procedural volume of bariatric endoscopy among early-career metabolic and bariatric surgeons. As diagnostic endoscopy, management of postoperative complications, and EBTs have become essential components of contemporary MBS, these findings emphasize the need for a standardized endoscopic training curriculum specifically designed for bariatric surgeons.

## Supplementary Information

Below is the link to the electronic supplementary material.

Supplementary file1 (DOCX 27 KB)

## Data Availability

No datasets were generated or analysed during the current study.

## Abbreviations

MBS: Metabolic and bariatric surgery
EBTs: Endoscopic bariatric therapies
IGB: Intragastric balloon
ESG: Endoscopic sleeve gastroplasty
TORe: Transoral outlet reduction
OR: Operative room
IOE: Intraoperative endoscopy
RYGB: Roux-en-Y gastric bypass
SG: Sleeve gastrectomy
