# Correction: Young-IFSO Endoscopy Training and Education Survey

**DOI:** 10.1007/s11695-025-07977-4

**Published:** 2025-06-13

**Authors:** Daniel Moritz Felsenreich, Anna Carolina Batista Dantas, Shahab Shahabi, Tamer N. Abdelbaki, Roxanna Zakeri, Mostafa E. Nagy, Daniel Gero, Elena Ruiz-Úcar, Zhi-Yong Dong, Wah Yang, Megan Jenkins, Sonja Chiappetta, on behalf of Young-IFSO Collaborative Group

**Affiliations:** 1 Division of Visceral Surgery, Department of General Surgery, Medical University of Vienna, Vienna, Austria; 2 Departamento de Cirurgia, Universidade Federal do Rio Grande do Norte (UFRN), Natal, Brazil; 3 Department of Surgery, Minimally Invasive Surgery Research Center, Division of Minimally Invasive and Bariatric Surgery, Iran University of Medical Sciences, Tehran, Islamic Republic of Iran; 4 Alexandria University Faculty of Medicine, Alexandria, Egypt; 5 Department of Upper GI Surgery, University College London Hospital NHS Foundation Trust, London, UK; 6 General Surgery Department, Ain Shams University, Cairo, Egypt; 7 Department of Surgery and Transplantation, University Hospital Zurich, University of Zurich, Zurich, Switzerland; 8 Metabolic and Endocrine Unit, General and Digestive Surgery Department, Fuenlabrada University Hospital, Rey Juan Carlos University, Madrid, Spain; 9 Department of Metabolic and Bariatric Surgery, The First Affiliated Hospital of Jinan University, Guangzhou, China; 10 Department of Surgery, Assistant Professor, NYU Langone Health, New York, NY USA; 11 Department of General Surgery, Center of Excellence Bariatric and Metabolic Surgery, Ospedale Evangelico Betania, Naples, Italy

**Correction: Obesity Surgery**

10.1007/s11695-025-07915-4

In the published article, the list of authors who are part of the Young-IFSO Collaborative Group is missing. They are as follows:

Ala Wafa

Carlo Gazia

Qasim Alhasnawy

Abd-Elfattah Kalmoush

Kalifi Maroin

Julio Adan Campos Badillo

Serena Esposito

Ahmed Abo Elmagd

Mohamed Mosaad kandel

Mohammed Atef Alokl

Mohamed H Zidan

Arianna Trizzino

Carlo Giove

Giorgio Ammerata

Giorgio Crocetti

Giovanna Berardi

Lorenzo Petagna

Antonio Vitiello

Beatrice Molteni

Michela Campanelli

Vincenzo Schiavone

Muhammad Ismail Yar Uttra

Agostino Fernicola

Salvatore Russo

Roberta Isernia

Eleonora Vichi

Andrea Quazzico

Simone Gargarella

Massimo Biondo

Nicolas Zucchini

Omar Ghazouani

Lorenza Beomonte Zobel

Massimiliano Casadei

Alessandra Iodice

Giovanni Tomasicchio

Cosimo Saviello

Rossella D'Alessio

Antonio Alvigi

Chiara De Lucia

Marta Silvestri

Georgios Geropoulos

Thomas Auguste

Alexandre Henrard

Reem Mohamed Korany

Osman Sibic

Amir H Ali

Efstratia Baili

Qiuye Cheng

Stephen Ka-Kei Ng

Gowsick Shanmuga Sundaram

Sameh Khaled Ahmed Ali Ibrahim

Ahmed Matar

Mahmoud Abdelbaky Mahmoud

Ahmed Nabil Elhoofy

Reza Karami

Oğuzhan Şal

Abanoub Awad

Yunusemre Tatlıdil

Adem Ozcan

Mohammad Ahmad Abd-erRazik

Rodrigo Dallegrave Corrêa Da Silva

Rodolfo Vasconcelos

Lucas Marques

Mohamed Alsayed Mohamed Farag Altager

Jianxin Chen

Hamed Gol Mohammadzadeh

Michael Gerges

Nariman Mehrnia

Ommolbanin Abed Firouzjah

Avallone

Amin Dalili

Barmak Gholizadeh

Ali Vahidirad

HuanLong Zhong

Sherko Abbas Nadir

Elyas Mostafapour

Alexandre Cenatti

Luiz Henrique Mestieri

Victor Pereira Chaves

Alex Craven

Ali Solouki

Pisica Radu-Mihai

Mohsen Pakzad

Saeed Madani

Mohammad Iranmanesh

Alireza Amirbeigi

Saeid shams Nosrati

Amir Hossein Davarpanah Jazi

Rossella Palma

Muhammad Umar Younis

G Balamurugan

Marius Nedlecu

Panagiotis Lainas

Yuki Oe

Thibaut Coste

Julio Adan Campos Badillo

Halit Eren Taskin

Ana Senent-Boza

Muhammad Umar

Christine Stier

Eulalia Ballester

Sjaak Pouwels

Anil Ergin

Napoleón Salgado Macías

Mariafelicia Valeriani

Alfonso Bosco

Nicoletta Basile

Paula Richwien

Lisa Gensthaler

Natalie Vock

Magdalena Mairinger

Cosimo Saviello

Omar Ghazouani

Daniel Reichhold

Jakob Eichelter

Vincenzo Schiavone

Georgios Karagiannidis

Yunus Yapalak

Vasileios Leivaditis

Ryland Samuel Stucke

Mehmet Buğra Bozan

Piotr Major

Simon Laplante

Massimo Biondo

Antonio Vitiello

Dimitri J Pournaras

Elisa Galfrascoli

Romano Schneider

Chiara Procaccini

Clarisa Birlog

Luca Ferraro

Maria Spagnuolo

Bassem Amr

Leo Brown

Andrea Quazzico

Andrew J Beamish

Ravikrishna Mamidanna

Muhammed Kadir YILDIRAK

Fatih Can Karaca

Ahmad Junaidi

Vincenzo Nicastro

Gabriela Smith

Chue Koy Min

De Capua Michele

Eman Mohammed Abdelsalam

Ke En Oh

Adisa Poljo

Marta Cuadrado-Ayuso

Mohamed H Zidan

Abd-Elfattah Kalmoush

Maria Lourdes Montero Alcaraz

Mahak Bhandari

Aishath Azna Ali

Ana Paulina Pimienta

Kayhan Ozdemir

Emilio Morales

Mohammed Atef Mohammed Alokl

Calabrese Pietro

Rob Snoekx

Agostino Fernicola

Georgios Ioannis Verras

Sercan Yuksel

Vasileios Mousafeiris

Edwin Vicente

Mert Tanal

Mandour O Mandour

Giorgi Zurabashvili

Francesk Mulita

Javier Lorenzo Pérez

Yaninee

Muhammed Rasid Aykota

Muhammed Ucuncu

Julio Adán Campos-Badillo

Adriana Marcela Zúñiga Rojas

Ellen Deleus

Adrian Gerard

Luigi Eduardo Conte

Claudia Meza Muñoz

Yuntao Nie

Hüseyin Güven Görkem

Mauro Andres Perdomo

Taryel Omarov

Ida Francesca Gallo

Rodrigue Chemaly

Andrew G Robertson

Elkin Eduardo Benítez Navarrete

Amanda Belluzzi

Ahmed A AlKhalifa

Enas Alqahtani

Patrick Téoule

Angelo Iossa

Natalia Dowgiałło-Gornowicz

Niccolo Petrucciani

Çağlar Ertekin

Eleni Amelia Felinska

Paul Cromwell

Midhat Abu Sneineh

Lidia Castagneto-Gissey

Mohamed Saleem Noormohamed

André Lázaro

Therese Reinstaller

Beniamino Pascotto

Abdullah Almunifi

Omar Hussein Ngotho

María José Dominguez

Dimitrios Kehagias

Manuel Ordonez

Ceyhun Aydogan

Turgay Yıldız

Giovanni Tomasicchio

Ahmed Barazanchi

Gianluca De Santo

İsmail Çalıkoğlu

Ozan Şen

Jonathan Sivakumar

Muzaffer Önder Öner

Adedire Timilehin Adenuga

Orkhan Verdiyev

Burhan Mayir

Hüseyin Eken

Tuna Bilecik

Xavier Sousa

Abdullah Sisik

Suleyman Caglar Ertekin

Kahei Au

Amr Elgazar

Jennifer M Klasen

Muhammad Haider Ali

Henrard Alexandre

Sherif Emad Abdelmawgoud

Nariman Mehrnia

Mohammad Fathi

Paola Dongo

Salvatore Tolone

Nader Moeinvaziri

Abdulellah Niyaz

Shakhawan Othman Qader

Elemawy mohamed

Suhaib Ahmad

Jhony Alejandro Delgado Salazar

Álex Morera-Grau

Daniel Leonard Chan

Chiara Isabella Miligi

Georgia Doulami

Nadia De Falco

Adam Abu-Abeid

Muhammed Taha Demirpolat

Fathi Elzowawi

Ahmed Abokhozima

