# Supplementary material for: Young-IFSO Endoscopy Training and Education Survey
Source: Obes Surg. 2025 Jun 3;35(7):2498–505. doi: 10.1007/s11695-025-07915-4 (PMC12271299; doi:10.1007/s11695-025-07915-4)
Supplement: Supplementary file 1 — Supplementary file1 (DOCX 27 KB) [file 11695_2025_7915_MOESM1_ESM.docx]

**Young IFSO Endoscopy Training and Education Survey**

| NR | QUESTIONS | ANSWERS |
| --- | --- | --- |
|  | **Basic Information** |  |
| 1 | Gender | Male |
|  |  | Female |
|  |  | Non-binary |
| 2 | Age |  |
| 3 | Nationality |  |
| 4 | Specialty | Surgeon |
|  |  | Endoscopist |
|  |  | Other |
| 5 | Current position | Student |
|  |  | Trainee |
|  |  | Consultant Surgeon |
|  |  | Consultant Endoscopist |
|  |  | Other |
| 6 | Is your center certified as a bariatric center of excellence? | Yes |
|  |  | No |
| 7 | Where are you working? | Private Hospital |
|  |  | Public Hospital |
|  |  | University Hospital |
|  |  | Private and Public/University |
|  |  | Military Hospital |
|  |  | Other |
| 8 | How many upper endoscopies (excl. ERCP) do you perform in total per year? | 0 |
|  |  | 1-50 |
|  |  | 51-100 |
|  |  | 101-500 |
|  |  | 501-1000 |
|  |  | More than 1000 |
| 9 | How many lower endoscopies do you perform in total per year? | 0 |
|  |  | 1-50 |
|  |  | 51-100 |
|  |  | 101-500 |
|  |  | 501-1000 |
|  |  | More than 1000 |
| 10 | How many routine endoscopies do you perform in total per year? | 0 |
|  |  | 1-50 |
|  |  | 51-100 |
|  |  | 101-500 |
|  |  | 501-1000 |
|  |  | More than 1000 |
| 11 | How many emergency endoscopies do you perform in total per year? | 0 |
|  |  | 1-50 |
|  |  | 51-100 |
|  |  | 101-500 |
|  |  | 501-1000 |
|  |  | More than 1000 |
| 12 | How many ERCP do you do perform in total per year? | 0 |
|  |  | 1-50 |
|  |  | 51-100 |
|  |  | 101-500 |
|  |  | 501-1000 |
|  |  | More than 1000 |
|  | **Endoscopic education** |  |
| 13 | Is endoscopic education integrated in the curriculum of General Surgery training in your nation (surgeons only)? | Yes |
|  |  | No |
| 14 | Have you performed endoscopic education during your training? | No |
|  |  | Yes, for ≤6 weeks |
|  |  | Yes, for ≤3 months |
|  |  | Yes, for ≤6 months |
|  |  | Yes, for more than 6 months |
| 15 | At which year of education have you performed your first endoscopy? | 1st |
|  |  | 2nd |
|  |  | 3rd |
|  |  | 4th |
|  |  | 5th |
|  |  | 6th |
|  |  | Not during Education |
| 16 | At which age have you performed your first endoscopy? |  |
| 17 | Estimate, how many endoscopies have you performed during your entire residency | 0 |
|  |  | 1-50 |
|  |  | 51-100 |
|  |  | 101-500 |
|  |  | 501-1000 |
|  |  | More than 1000 |
| 18 | Have you attended a course for upper endoscopy training? | Yes |
|  |  | No |
| 19 | What types of endoscopic training is available in your institution? | Cadaver course |
|  |  | Animal course |
|  |  | Dry lab |
|  |  | Simulation |
|  |  | None of above |
|  |  | Other |
| 20 | Are you interested in attending upper endoscopy training courses? | Yes |
|  |  | No |
|  | **Diagnostic and complication management** |  |
| 21 | Do you perform diagnostic upper endoscopy prior to primary bariatric surgery on your own? | Yes |
|  |  | No |
| 22 | Do you perform diagnostic upper endoscopy prior to revisional bariatric surgery on your own? | Yes |
|  |  | No |
| 23 | Do you perform diagnostic upper endoscopy during bariatric surgery on your own? | Yes |
|  |  | No |
| 24 | Do you perform diagnostic upper endoscopy in suspected complication (bleeding, leakage) on your own? | Yes |
|  |  | No |
| 25 | Is endoscopic complication management performed in your hospital? | Yes |
|  |  | No |
| 26 | Do you personally perform endoscopic complication management in your hospital? | Yes |
|  |  | No |
| 27 | Which of the endoscopic techniques for complication management are you confident with and perform on your own? | Intraluminal Bleeding Management |
|  |  | Stent Placement |
|  |  | Pigtail Placement |
|  |  | Endoscopic placement of a feeding tube |
|  |  | Pneumatic dilatation |
|  |  | Endoscopic vacuum therapy |
|  |  | None of above |
|  |  | Other |
| 28 | Do you have an endoscopy tower in the Operating Room? | Yes |
|  |  | No |
| 29 | Do you perform endoscopic evaluation (leakage test, staple line bleeding, etc.) at the end of MBS? | Yes |
|  |  | No |
| 30 | Are endoscopies for MBS in your center more commonly performed by | Surgeons |
|  |  | Endoscopists |
|  | **Endoscopic Bariatric Therapy (EBT)** |  |
| 31 | Is EBT performed in your hospital? | Yes |
|  |  | No |
| 32 | Do you personally indicate EBT? | Yes |
|  |  | No |
| 33 | Is intragastric balloon placement performed in your hospital | Yes |
|  |  | No |
| 34 | Is endoscopic sleeve gastroplasty performed in your hospital? | Yes |
|  |  | No |
| 35 | Is transoral outlet reduction (TORe) after RYGB performed in your hospital? | Yes |
|  |  | No |
| 36 | Do you perform EBT by yourself? | Yes |
|  |  | No |
| 37 | Do you perform endoscopic intragastric balloon placement by yourself? | Yes |
|  |  | No |
| 38 | Do you perform endoscopic sleeve gastroplasty by yourself? | Yes |
|  |  | No |
| 39 | Do you perform transoral outlet reduction (TORe) after RYGB by yourself? | Yes |
|  |  | No |
| 40 | Would you like to be listed as collaborative author of this survey? | Yes |
|  |  | No |
